# Diseases Admitted under the Care of the Physicians of the Westminster Hospital, from the 22d of October to the 26th of November, 1800

**Published:** 1801-01

**Authors:** 


*Difeafes admitted under the care of the Phyficians of the Weftminfter Hofpital, from the 22d of October to the 26th of November,* 1800.

| | No. of Cafes. | | No. of Cafes. |
|---|---|---|---|
| Continued Fever | 24 | Hæmoptyfis | 1 |
| Intermittent | 1 | Hyfteria | 3 |
| Pleurify | 2 | Itch | 7 |
| Amenorrhœa | 2 | Lichen | 1 |
| Anafarca | 2 | Lithiafis | 1 |
| Apoplexia | 1 | Lumbago | 1 |
| Afcites | 2 | Obftipatio | 1 |
| Catarrh | 8 | Paralyfis | 2 |
| Cholera | 1 | Phthifis | 1 |
| Cough, &c. | 14 | Pleurodyne | 1 |
| Diarrhœa | 3 | Rheumatifm | 9 |
| Dyfpnœa | 4 | Syphilis | 2 |
| Enterodynia | 2 | Vertigo | 2 |
| Epilepfy | 1 | Vomitio | 1 |
| Erythema | 1 | Worms | 1 |
| Gaftrodynia | 1 | | |

